# Thickness Dispersion of Surface Plasmon of Ag Nano-thin Films: Determination by Ellipsometry Iterated with Transmittance Method

**DOI:** 10.1038/srep09279

**Published:** 2015-03-23

**Authors:** Junbo Gong, Rucheng Dai, Zhongping Wang, Zengming Zhang

**Affiliations:** 1 Department of Physics, University of Science and Technology of China, Hefei, Anhui 230026, China; 2 The Centre of Physical Experiments, University of Science and Technology of China, Hefei, Anhui 230026, China

## Abstract

Effective optical constants of Ag thin films are precisely determined with effective thickness simultaneously by using an ellipsometry iterated with transmittance method. Unlike the bulk optical constants in Palik's database the effective optical constants of ultrathin Ag films are found to strongly depend on the thickness. According to the optical data two branches of thickness dispersion of surface plasmon energy are derived and agreed with theoretical predication. The thickness dispersion of bulk plasmon is also observed. The influence of substrate on surface plasmon is verified for the first time by using ellipsometry. The thickness dependent effective energy loss function is thus obtained based on this optical method for Ag ultrathin films. This method is also applicable to other ultrathin films and can be used to establish an effective optical database for ultrathin films.

Surface plasmon, as a collective electron oscillation behavior in surface mode, has been extensively studied since the prediction by Ritchie^1^. The most common plasmonic material, silver, due to its wide optical and electrical application, has received amount of attention while there is still something obscure especially for ultrathin silver films^2^,^3^,^4^,^5^. For example, thickness dependence of surface plasmon of ultrathin films which was predicted in 1957 lacks straightforward optical experimental evidence ever since. The optical and dielectric properties of ultrathin silver films have significant difference from bulk material. One way to analyze surface plasmon is through energy loss function which can be deduced directly from optical constants, refractive index *n* and extinction coefficient *k*^6^,^7^. Generally, optical constants can be obtained either by optical methods^8^ or by transmission/reflection electron energy loss spectroscopy (TEELS/REELS) techniques^8^,^9^. However, Palik's optical database in common use actually is not applicable to ultrathin films in thickness under several tens nanometers considering that it has been proved by many researchers that the optical constants of a film are dependent on thickness when the film is very thin^10^,^11^. This limitation has caused difficulty to surface analysis area as theorists working on surface electron spectroscopy have found that the simulation results with Palik's optical data can describe bulk material while have an obvious deviation with experimental results of thin films^12^. In fact, either optical or REELS measurements were almost all performed on single crystal Ag surface or bulk Ag^9^. However, actual applications based on surface plasmon generally use thin Ag films made of nanoparticles^13^. Study on ultrathin Ag film is significant for exploring the properties of thin film plasmon^14^,^15^.

To achieve this purpose, firstly, the optical constants of ultrathin films need to be determined precisely. Even for better optical coating performance, it is necessary to study the thickness dependence of optical constants to extend the optical database from bulk to 2-dimension film; this requires the simultaneous determination of optical constants with film thickness. However, the extraction of optical data for ultrathin films from REELS spectra has not yet been performed because of the analysis difficulty and the need of clean thin film surface prepared *in situ*^2^. Therefore, the optical method should be more practically useful. Ellipsometry, as the most effective optical method to obtain optical constants, also faces a challenge when applied to ultrathin absorbing films. The traditional ellipsometry usually measures the change of amplitude and phase, i.e. tanΨ(*λ*) and Δ(*λ*) respectively, of the polarized light of wavelength *λ* after reflection from the sample surface. The relationship between the ellipsometry parameters is *R_p_*/*R_s_* = tanΨ·exp(*i*Δ), where *R_p_* and *R_s_* are the complex reflection coefficients of polarized *p*- and *s*-waves, respectively, which are related with film thickness and optical constants by Fresnel equation. Solution of this complex equation (in total number of 2*N* where *N* is the number of wavelength spectrum channels) requires one of the quantities, i.e. film thickness *d* and optical constants (*n*(*λ*) and *k*(*λ*)), is known. When film thickness and optical constants, in total number of 2*N* + 1, are all unknown as in the present case, it is impossible to precisely determine them simultaneously due to lack of sufficient constraint equations. In fact, the non-uniqueness of solutions is the main problem with traditional ellipsometry. Therefore, a dispersion relationship for the dependence of optical constants on wavelength is usually assumed. However, there is no any suitable dispersion model for ultrathin metal films.

## Results

**Figure 1:**
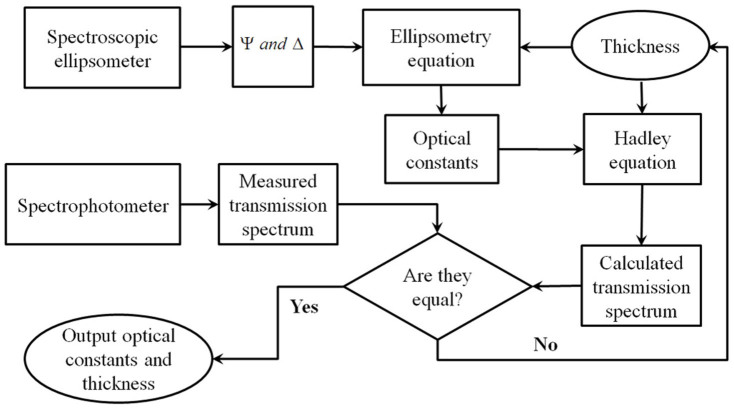
Schematic of ellipsometry iterated with transmittance method.

**Figure 2:**
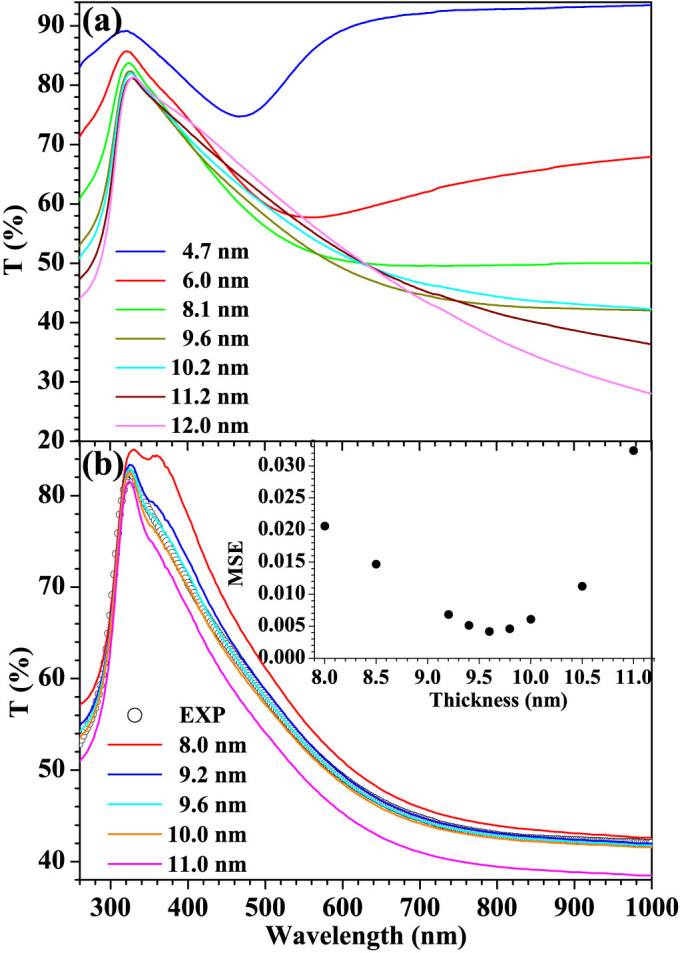
(a) Measured transmittance spectra for ultrathin Ag film on quartz glass substrate samples at different effective film thickness; (b) Fitted transmittance spectra of 9.6 nm thick film assuming different thickness values (inset shows the corresponding MSE values of fitting results).

In this paper, an ellipsometry iterated with transmittance method is proposed and has been used to determine effective optical constants of nano-thin films. The basic consideration is to supply additional *N* constrained equations with the help of the measured transmittance spectrum *T*(*λ*). The flow chart of this method is shown in Fig. 1. First, with an initial thickness value assumed and the measured ellipsometric parameters, Ψ and Δ, the initial optical constants are obtained based on the ellipsometry equation. Then a transmittance spectrum is computed by using this initial thickness and the optical constants based on Hadley equation (Supplementary information). The calculated transmittance spectrum *T*_cal_(*λ*) is then compared with the measured spectrum *T*_exp_(*λ*). Here we use mean square error (MSE) to quantify the difference,
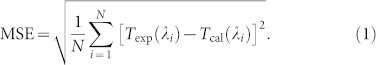


The thickness value is then altered to minimize MSE. The iteration procedure is performed until a minimum MSE value is achieved, indicating that the final derived thickness value and optical constants altogether satisfy the ellipsometry equation and Hadley equation. Fig. 2(a) shows the measured transmittance spectra for ultrathin Ag film on quartz glass substrate samples at different effective film thicknesses whose values are obtained by above procedure. As an example shown by Fig. 2(b), the calculated spectrum is best matched with the experimental spectrum for the effective thickness value of 9.6 nm corresponding to minimum MSE. The self-consistent optical constants and film thickness can thus be precisely and simultaneously determined. Unlike the traditional ellipsometry dispersion model is not necessary here. The resultant optical constants are unique.

However, it is necessary to point out that this method is based on the assumption of a uniform film on a substrate. The dielectric response of nanostructured materials to an external perturbation depends on significantly the sample morphology as boundary conditions of Maxwell's equation. For an ultrathin metal film in the form of metal islands on a substrate, the dielectric property is in fact that of the mixture of the islands and the embedding dielectric (the air here) and is hence connected with the surface roughness. To simplify the problem a concept of effective optical constants has been introduced within the limits of a homogeneous layer model^16^. Theoretical investigations have also treated heterogeneous media as homogeneous with some effective optical properties^17^,^18^. Here, we define an effective thickness and effective optical constants which can satisfy ellipsometry equation and Hadley equation simultaneously for ultrathin metal film in form of islands. In addition, considering that the size of optical beam in millimeters is much larger than that of islands, the film can be regarded as homogeneous on a macro-scale and thus the film properties can be described by the effective optical constants of the homogeneous layer. The same values of effective optical constants and of effective thickness measured at different positions of the sample also confirm that this assumption is suitable.

**Figure 3:**
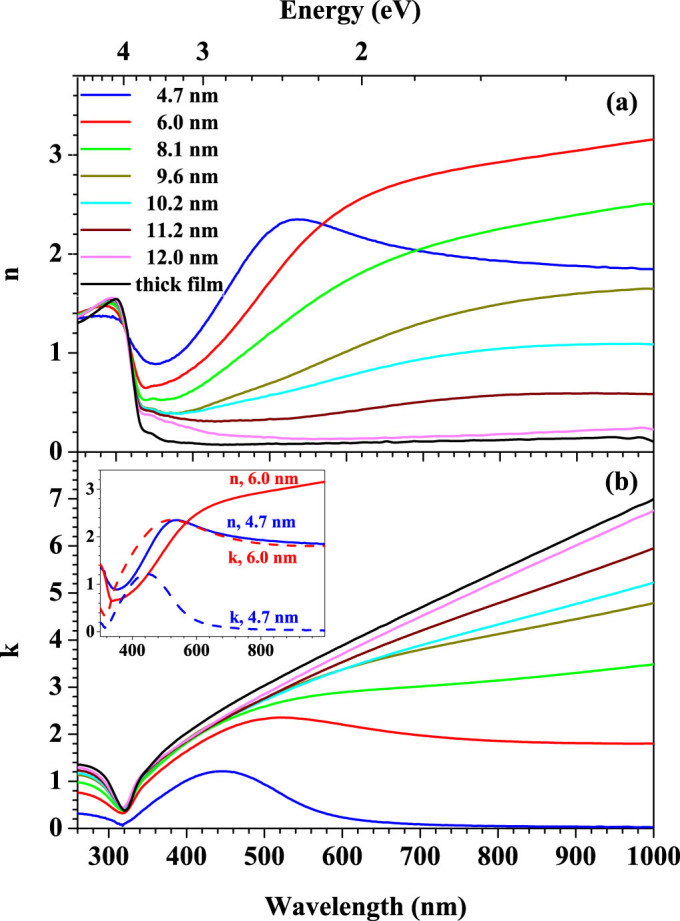
Dependence of the refractive index *n* (a) and extinction coefficient *k* (b) on the wavelength for silver films at different thicknesses. Inset in Fig. (b) shows the optical constants *n* and *k* of films with 4.7 nm and 6.0 nm effective thickness.

Fig. 3 shows the obtained refractive index *n*(*λ*) and extinction coefficient *k*(*λ*) in the wavelength range from ultraviolet to near infrared for silver ultrathin films at different thicknesses. The film with the thickness below 8 nm displays a non-metallic behavior considering that the value of refractive index is larger than that of extinction coefficient in wavelength range from 600 nm to 1000 nm (inset of Fig. 3). With increasing thickness the film changes to metallic behavior; the refractive index in range of 600-1000 nm gets larger at first from 4.7 nm thick and goes down from 6 nm thick and finally tends to be stable. Meanwhile the extinction coefficient monotonously increases with thickness while the increment gradually gets smaller. When the thickness is larger than 12 nm, both *n* and *k* tend to be stable and become closer to those of bulk Ag.

## Discussion

**Figure 4:**
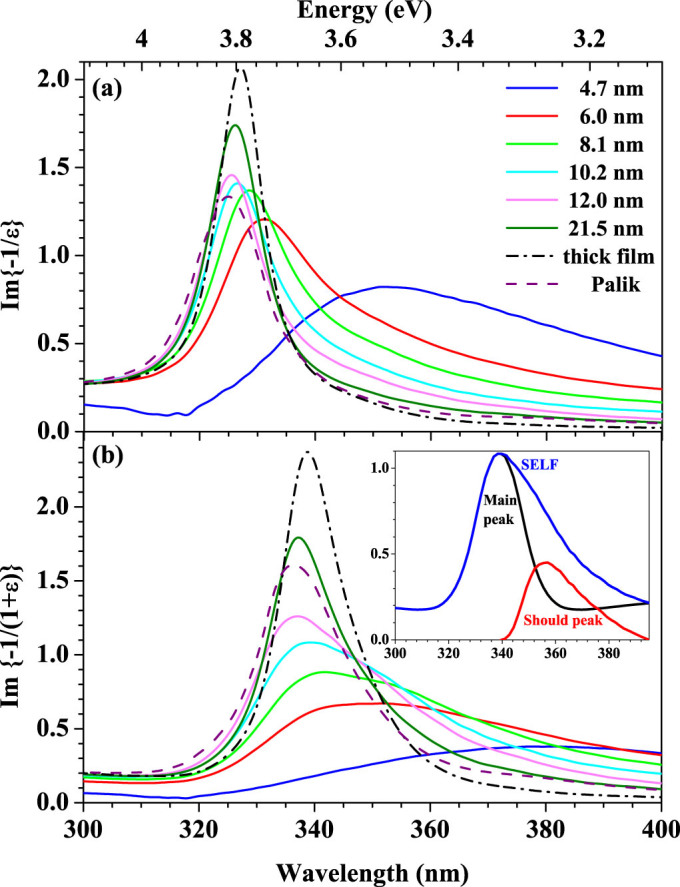
The bulk energy loss function (a) and surface and energy loss function (b) in ultraviolet region for silver films at different thicknesses. Inset in Fig. (b) shows the determination of shoulder peak by subtracting the symmetric main peak.

In REELS analysis the bulk energy loss function (BELF), 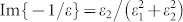, and the surface energy loss function (SELF), 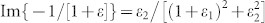, characterize the probability of electrons to undergo bulk excitation in volume and surface excitation in surface region, respectively^19^, where *ε*_1_ and *ε*_2_ are the real and imaginary parts of dielectric constant *ε*. Figs. 4(a) and 4(b) display the plots of effective BELF and effective SELF, derived from the present effective optical constants, of Ag films as a function of the photon energy in ultraviolet region, which respectively show the bulk plasmon excitation peak and surface plasmon peak. The peak positions of the BELF and SELF for a thick film, which can be seen as bulk Ag for ellipsometry, are 327 nm (3.79 eV) and 338 nm (3.67 eV), respectively. For comparison, the corresponding bulk and surface plasmon energies of bulk material derived from Palik's database are, respectively, 3.81 and 3.68 eV^8^.

**Figure 5:**
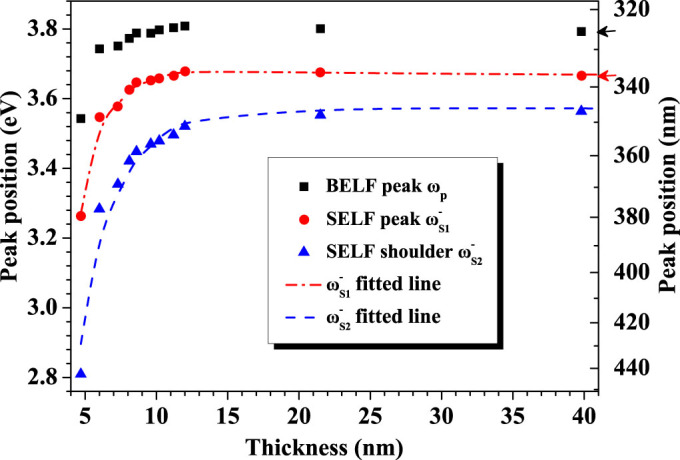
Thickness dependence of bulk plasmon and surface plasmon energies for silver films. (The red and blue fitted line represent the 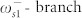 for metal-to-air interface and 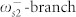 for metal-to-substrate interface, respectively. The red arrow marked the position of silver surface plasmon from Palik's database and the black one corresponding to volume plasmon.)

The thickness dependence of peak positions of BELF and SELF is shown in Fig. 5. It is obvious that both of them shift to a higher energy side with increasing thickness. While the thickness dispersion of surface plasmon can be well explained by the theory, the thickness dependence of bulk plasmon energy reflects the fact that the optical constants of thin films contain information of electronic structure of films, which is different from that of bulk sample. The bulk plasmon frequency can be expressed in terms of the free electron density (*ρ*), the electron charge (*e*) and effective mass (m*): 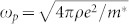. Similar to the observation for indium thin film case^20^, by increasing the film thickness the free electron density increases to reach the value of bulk silver, which agrees with the observed increasing metallic behavior with thickness as deduced from the optical constants shown in Fig. 3.

It can be seen from SELF in Fig. 4(b) that there is an obvious shoulder at lower energy side beside the main surface plasmon peak. The position of the shoulder is determined by subtracting the symmetric main peak as shown in the inset of Fig. 4(b). The main peak corresponds to surface plasmon at metal-to-air interface while the shoulder peak to the metal-to-substrate interface. The observation of shoulder peak provides a strong evidence for the existence of symmetric mode contributing from quartz glass substrate in ultrathin Ag films while the asymmetric mode is too weak to observe. Previous studies shows that the plasmon waves at the top and bottom surfaces of a film generate two different normal modes of higher and lower frequencies^21^. In the case of a free-electron metal film surrounded by a dielectric *ε_a_*, the theoretical surface plasmon dispersion gives^1^,
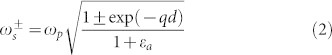
where *d* is the thickness of a film and *q* the wave vector. For small *qd*, only the 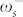 branch can exist^22^. In the present case, the metal film is surrounded by air (*ε_a_* = 1) and quartz glass (*ε_a_* = 2.3) by two sides; we assign the 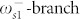 for metal-to-air interface and 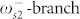 for metal-to-substrate interface. The thickness dispersion is fitted for two branches as, 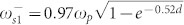 and 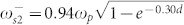 where *d* is in nm, respectively. Quantitative difference of the observed surface plasmon energy with Eq. (2) is due mainly to non-free-electron-like behavior of silver film.

In fact, the film surface is not an ideally flat plane but made of particle islands as observed by atomic force microscopy (AFM) (Supplementary information, Figure S2). Therefore the above experimental result may also be qualitatively explained by another theoretical result^23^. AFM images indicate that the particle size relates with the thickness, and the van der Drift model presents the proportional increasing behavior^24^,^25^. On the other hand, considering an isolated spherical particle (relative permittivity *ε_r_*) surrounded by a dielectric medium of *ε_a_*, the surface resonance condition is given by 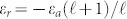, where 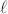 is an integer^23^. For a free-electron-like metal, the surface plasmon frequency is 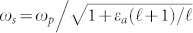. Here, the 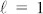 is related to dipole mode oscillation, while 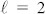 corresponds to the quadrupole oscillation, and so on. The resonance at lowest frequency corresponding to 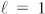 dominates in very small particles. However, for larger nanoparticles where the dipole approximation is no longer valid, the resonance mode depends explicitly on the particle size^26^. The larger the particles become, the more important the higher order modes are because the electromagnetic field can no longer polarize the nanoparticles homogeneously as the particle size approach to the wavelength of the exciting radiation. As a conclusion, with increasing particle size the resonance frequency at higher order modes increases gradually; this results in the thickness dispersion of plasmon energy towards the value of bulk material for a thick film.

In conclusion, employing the ellipsometry iterated with transmittance method, the effective thickness and effective optical constants of thin Ag films deposited on quartz glass have been simultaneously obtained. The derived effective surface energy loss function and bulk energy loss function of Ag nano-thin films with different thicknesses have shown an obvious difference with that of Palik's data for single crystal silver. Thickness effect on the energy loss function has been discussed and the influence of substrate on surface plasmon energy predicted by Ritchie has also been observed. This optical measurement method based on ellipsometry is helpful to study effective energy loss function and plasmon properties of ultrathin films.

## Methods

Ag of 99.99% purity was deposited on pre-cleaned quartz glass substrate to prepare the silver thin films by AC magnetron sputtering. The transmittance spectra from 260 nm to 1000 nm of the prepared Ag films were measured at normal incidence by a double-beam spectrophotometer (Shimazu Co., Inc. SolidSpec-3700). Ellipsometric spectra from 260 nm to 1000 nm were collected at incidence angles of 58° and 68° by a rotating-polarizer ellipsometer (J.A. Woollam Co., Inc. M-2000U). The ellipsometric spectra at two incident angles were used simultaneously in fitting ellipsometric parameters, Ψ and Δ.

## Author Contributions

J.B.G. and Z.M.Z. conceived and designed the experiments. J.B.G. prepared the figures and wrote the manuscript with the assistance from all authors. R.C.D. and Z.P.W. participated in supervising the work. All authors reviewed the manuscript.

## Supplementary Material

Supplementary Information Supplementary infromation
