# Supplementary material for: Thickness Dispersion of Surface Plasmon of Ag Nano-thin Films: Determination by Ellipsometry Iterated with Transmittance Method
Source: Sci Rep. 2015 Mar 23;5:9279. doi: 10.1038/srep09279 (PMC4369689; doi:10.1038/srep09279)
Supplement: Supplementary Information — Supplementary infromation [file srep09279-s1.pdf]

Thickness Dispersion of Surface Plasmon of Ag Nano-thin Films: Determination by Ellipsometry Iterated with Transmittance Method

Junbo Gong[1], Rucheng Dai[2], Zhongping Wang[2] and Zengming Zhang[2*]

[1]*Department of Physics, University of Science and Technology of China, Hefei, Anhui 230026, China*

[2]*The Centre of Physical Experiments, University of Science and Technology of China, Hefei, Anhui 230026, China*

*Corresponding author: zzm@ustc.edu.cn

Ellipsometry spectra

Fig. S1. Shows the typical fitted ellipsometry spectra for ellipsometric parameters Ψ and Δ; the calculated parameters match very well with the measured data.

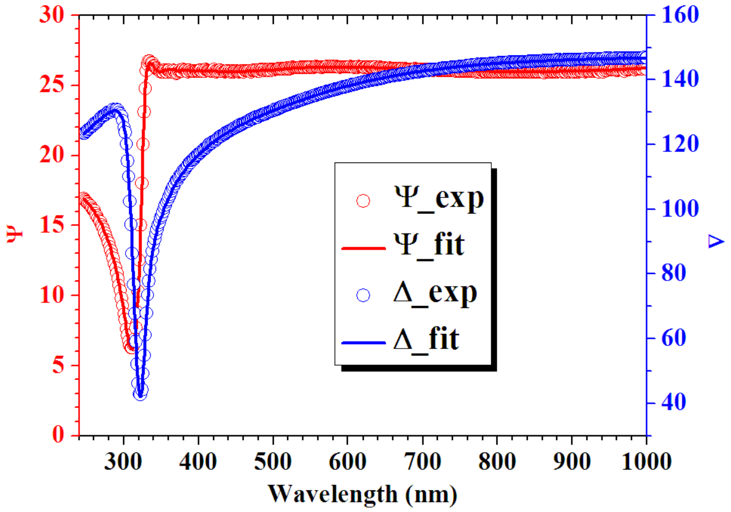

Figure S1. Ellipsometric parameter fitting spectra for 9.6 nm silver film at 58° incident angle.

Hadley equation

The transmittance spectrum was calculated according to Hadley equation:[1]

$$R = \frac{A_1 \cosh \alpha + A_2 \sinh \alpha - A_3 \cos \beta + A_4 \sin \beta}{B_1 \cosh \alpha + B_2 \sinh \alpha - B_3 \cos \beta + B_4 \sin \beta} \tag{1}$$

$$T = \frac{8n_g \left(n^2 + k^2\right)}{B_1 \cosh \alpha + B_2 \sinh \alpha - B_3 \cos \beta + B_4 \sin \beta} \tag{2}$$

where R and T represent respectively the reflectance and transmittance when light is incident onto substrate from a film. n and k are respectively the real and imaginary parts of the complex refractive index of the reflecting material, i.e. the film; n_g is the refractive index of the substrate. Here,

$$\alpha = 4\pi kd/\lambda, \quad \beta = 4\pi nd/\lambda; \tag{3}$$

$$A_1 = \left(n^2 + k^2 + 1\right)\left(n^2 + k^2 + n_g^2\right) - 4n^2 n_g, \tag{4}$$

$$A_2 = 2n\left[n_g \left(n^2 + k^2 + 1\right) - \left(n^2 + k^2 + n_g^2\right)\right], \tag{5}$$

$$A_3 = \left(n^2 + k^2 - 1\right)\left(n^2 + k^2 - n_g^2\right) + 4k^2 n_g, \tag{6}$$

$$A_4 = 2k\left[n_g \left(n^2 + k^2 - 1\right) - \left(n^2 + k^2 - n_g^2\right)\right], \tag{7}$$

$$B_1 = \left(n^2 + k^2 + 1\right)\left(n^2 + k^2 + n_g^2\right) + 4n^2 n_g, \tag{8}$$

$$B_2 = 2n\left[n_g \left(n^2 + k^2 + 1\right) + \left(n^2 + k^2 + n_g^2\right)\right], \tag{9}$$

$$B_3 = \left(n^2 + k^2 - 1\right)\left(n^2 + k^2 - n_g^2\right) - 4k^2 n_g, \tag{10}$$

$$B_4 = 2k\left[n_g \left(n^2 + k^2 - 1\right) + \left(n^2 + k^2 - n_g^2\right)\right]. \tag{11}$$

Considering a system of metal film deposited on a fused silica wafer, its total transmittance T_c can be expressed as

$$T_c = \frac{TT_0}{1 - R_0 R_1} \tag{12}$$

where

$$R_0 = \left[\left(1 - n_g\right)/\left(1 + n_g\right)\right]^2 \tag{12}$$

$$T_0 = 1 - R_0 \tag{13}$$

$$R_1 = \frac{A_1 \cosh\alpha + A_2 \sinh\alpha - A_3 \cos\beta + A_4 \sin\beta}{B_1 \cosh\alpha + B_2 \sinh\alpha - B_3 \cos\beta + B_4 \sin\beta} \tag{14}$$

where R_0 and T_0 are reflectance and transmittance from the interface between vacuum and substrate respectively. R_1 is reflectance of the interface between the film and the substrate.

Surface topography analysis

To explore the influence of surface particle size on film properties, the surface morphology information was collected using atomic force microscopy (AFM) in the tapping mode (SII NanoTechnology Inc. NanoCute). AFM images in Figure S2 shows the surface morphology of Ag thin films with different thicknesses. They clearly indicate that all thin films are made of nanoparticles.

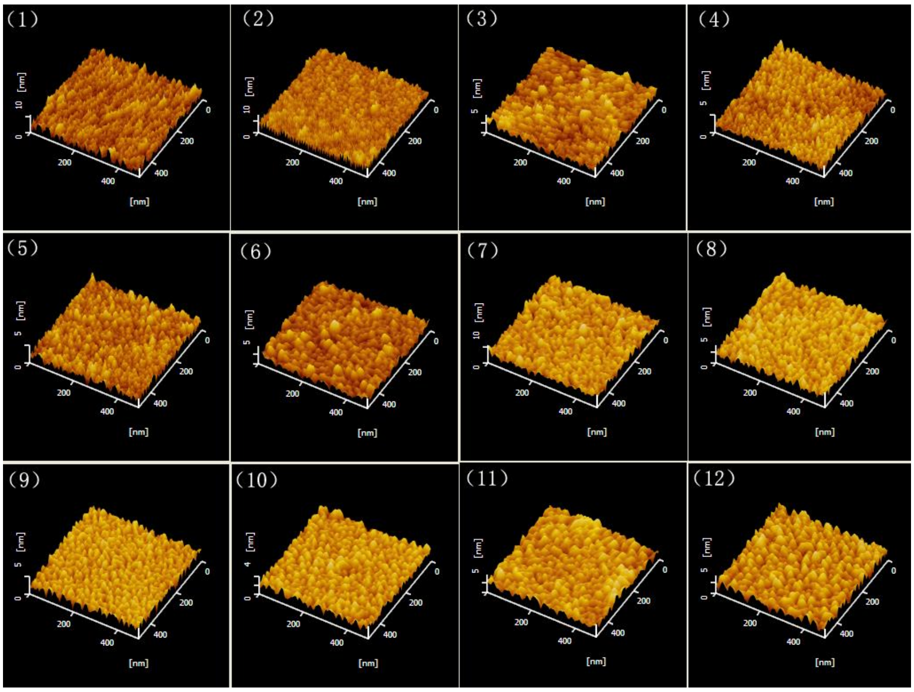

Figure S2. AFM topographic images (500 nm×500 nm) of the Ag films for various thickness values (in nm) from (1)-(12): 4.7, 6.0, 7.3, 8.1, 8.6, 9.6, 10.2, 11.2, 12.0, 21.3, 39.8, thick film.

Figure S3 presents the particle size (*D*) obtained from AFM images varying with the thickness (*d*) of thin films. It is seen that the particle size becomes larger as the thickness of thin film increases and the relationship is fitted by $D = 8.41 \times d^{0.39}$. This growing characteristic is explained by the van der Drift model.[2] At the initial stage of deposition, Ag atoms formed the random orientation nuclei. At the next competitive growth stage, the small particles aggregate to form larger ones keeping the same structure. In a word, under the same preparation conditions, the surface topography of thin film is dependent on film thickness.

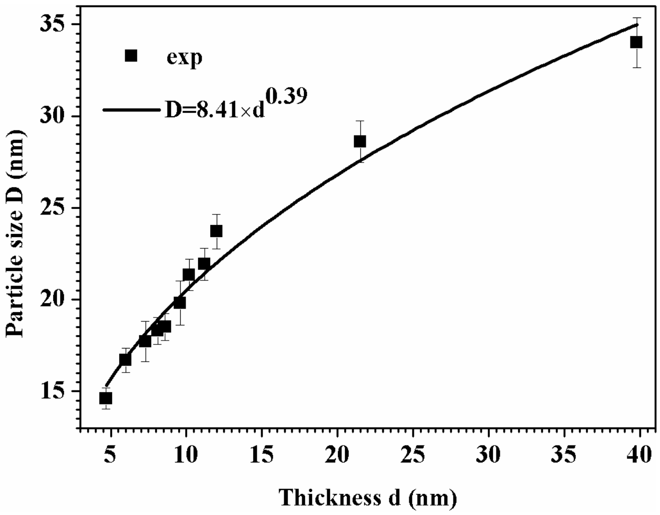

Figure S3. Variation of particle size (*D*) with film thickness (*d*); the curve is the linear fit.

1. Dwight, E. G., *American institute of physics handbook.* (Mcgraw-Hill, New York, 1963).

2. Vanderdr, A., Evolutionary selection a principle governing growth orientation in vapour-deposited layers. *Philips Research Reports*, **22**, 267 (1967).
